# Global patterns, biases, and advances in phylogeographic and genetic structure studies in Drosophilidae (Insecta: Diptera)

**DOI:** 10.1590/1678-4685-GMB-2025-0246

**Published:** 2026-02-09

**Authors:** Henrique R. M. Antoniolli, Maríndia Deprá, Lizandra Jaqueline Robe

**Affiliations:** 1 Universidade Federal do Rio Grande do Sul (UFRGS), Instituto de Biociências, Programa de Pós-Graduação em Genética e Biologia Molecular, Porto Alegre, RS, Brazil.; 2 Universidade Federal de Santa Maria (UFSM), Programa de Pós-Graduação em Biodiversidade Animal, Santa Maria, RS, Brazil.

**Keywords:** Drosophila, model species, molecular clock, molecular markers, phylogeography

## Abstract

Phylogeographic and genetic structure studies involving Drosophilids have clarified numerous processes that have shaped the evolution of biodiversity over time. In this review, we aim to (i) assess the main biases, gaps, and advances in the scientific literature on this topic; (ii) synthesize the major findings emerging from these studies; and (iii) identify congruencies and discrepancies in the phylogeographical histories of different species and regions. To achieve these goals, we conducted a comprehensive review of peer-reviewed literature on phylogeographic and genetic structure studies of Drosophilidae published between 1987 and 2024. After identifying and filtering relevant studies, we extracted and analyzed key information related to each topic. Overall, we have detected a straightforward predominance of studies involving species of the *Drosophila* genus, especially within the *melanogaster*, *obscura*, and *repleta* groups. Interestingly, most studies employed nuclear DNA markers, either alone or in combination with mitochondrial markers, and were conducted across more than one biogeographical region, primarily in the Palearctic and Nearctic. Thus, our synthesis underscores the importance of broader taxonomic sampling and increased attention to understudied regions to enhance our understanding of biodiversity dynamics in response to environmental changes at both local and global scales.

## Introduction

Phylogeography emerged as a new discipline nearly 40 years ago, when Avise et al. (1987) highlighted the lack of a field of study that connects population genetics with phylogenetic systematics. The term “phylogeography” was later formalized to describe research that integrates genealogies and biogeography across temporal and spatial dimensions, emphasizing historical factors that explain present-day distributions of lineages (Avise, 2009). In this way, it aims to evaluate patterns of distribution of genetic diversity across the species’ full geographic range, in contrast to studies that assess population structure among individual populations representing only part of the species’ overall range. Nevertheless, both approaches can substantially contribute to understanding the processes that have shaped the evolutionary history of the focal species, at global or more local scales. Moreover, when applied in a comparative framework, they may help address questions about the origin and evolution of biodiversity in specific biogeographical contexts.

Notably, these approaches have been applied to issues in speciation and conservation biology across various taxa, including animals (Garcez et al., 2018; Kjartanson et al., 2023) and plants (Zhao et al., 2019). They also contribute to current assessments of diversity and conservation by enabling the discovery of new species or lineages (Alfaro et al., 2018) and the accurate assignment of species distributions (Carvalho et al., 2023). Additionally, they may contribute to understanding ecological (Bonebrake et al., 2010) and evolutionary relationships (Rosauer et al., 2016) among populations or species, insights that are critical for explaining speciation, diversification, and the factors influencing survival and extinction. Finally, they may shed light on the general patterns of genetic diversity [such as intra- and interpopulation structure (Eizirik et al., 2001) and underlying demographic history (Zhang et al., 2017)], and help uncover historical processes that have shaped biodiversity [such as vicariance or fragmentation into refugia (Song et al., 2018), range expansion and secondary contact (Gum et al., 2005), introgression (Jeratthitikul et al., 2013) and coevolution (Cuthill and Charleston, 2015)]. 

To accomplish all these tasks, suitable molecular markers should be carefully selected. Ideally, they should exhibit several properties that maximize their informative power, including sufficient variability without saturation, reliable orthology assignment, and clocklike evolution, combined with technical practicality (i.e. their characterization should be scalable, cost-effective, and reproducible). They may also vary in their inheritance patterns, which contrasts maternally inherited mitochondrial DNA (mtDNA) with biparental nuclear DNA (nDNA) markers, as well as dominant and co-dominant markers [such as microsatellites (satDNA) and Single Nucleotide Polymorphisms (SNPs)]. Mitochondrial genes and satDNA have the advantage of faster mutation rates than nuclear genes, allowing us to infer relatively recent evolutionary events (Avise, 2004). On the other hand, nDNA exhibit biparental inheritance, providing a more comprehensive view of population genetic patterns and offering insights into long-term evolutionary processes. Historically, mtDNA has been widely used as the preferred marker for phylogeographic and population structure analyses (Beheregaray, 2008; Turchetto-Zolet et al., 2013), although marker choice may differ among taxa.

Drosophilidae is a diverse and broadly distributed family of Diptera, comprising more than 4,000 species that inhabit environments ranging from tundra to tropical regions (Bächli, 2025; Throckmorton, 1975) and exploit a wide range of substrates (Carson, 1971; Powell, 1997). The family is subdivided into 77 extant genera distributed across distinct lineages within the Drosophilidae phylogeny (Kim et al., 2024). In several cases, this arrangement results in the paraphyly of *Drosophila*, the “most famous” genus of the family. In fact, different *Drosophila* species, particularly *D. melanogaster*, have been widely used as model organisms across biomedical, ecological, and evolutionary research (Kohler, 1993; Markow and O’Grady, 2006). Notably, eight Nobel Prizes have been awarded to scientists for discoveries using *Drosophila* (Clancy et al., 2023). Their short generation time, ease of laboratory culture, and well-characterized genetics make them ideal candidates for such investigations. In the field of phylogeography, Drosophilidae species have contributed to understanding both shared and idiosyncratic evolutionary histories (Hurtado et al., 2004; Franco and Manfrin, 2013; De Ré et al., 2014; Yukilevich et al., 2018) and to assessing general diversification patterns (Markow, 2019).

In this paper, we reviewed the scientific literature on studies of phylogeography and genetic structure in Drosophilidae species, aiming to identify biases, gaps, and recent advances, and to synthesize the main findings and highlight congruences and discrepancies in phylogeographic patterns across biogeographic regions. For this task, we first identified and filtered our dataset. We then summarized each study according to its main characteristics, including the identity of the focal taxa, sampling design, molecular markers employed, main findings, and general implications. This allowed us to provide a comprehensive overview of the phylogeographic landscape of Drosophilidae for each biogeographic region.

## Material and Methods

We conducted the literature surveys in March 2025, in the Web of Science (Clarivate Analytics) database, searching for papers published from 1987 to 2024 using the string TS=(drosophil*) AND TS=(“phylogeograph*” OR “population structure” OR “genetic structure” OR “genetic diversity” OR “demographic history” OR biogeograph* OR diversification OR dispersal OR colonization), that were searched in the titles, abstracts and keywords. We then filtered the initial set of results to include only original papers.

We employed the ASReview tool (van de Schoot *et al.*, 2021), a machine learning-aided pipeline that applies active learning, for an initial automated screening. To train the model, we first reviewed the titles and abstracts of a random set of 200 articles, labeling each paper as either “relevant” or “non-relevant” to our search focus. The ASReview pipeline then prioritized the remaining studies based on their predicted likelihood of relevance. Screening was stopped after 300 consecutive non-relevant records, when we had already manually inspected 2,050 of the 3,602 papers. ASReview input and output files are presented in the Supplementary Material (Tables S1 and S2, respectively). 

After recording the papers selected by ASReview in a Microsoft Excel spreadsheet, we extracted basic information from each study, as follows: (i) year and journal of publication; (ii) number and identity of species and species group(s) evaluated in the study, following the taxonomic data available on the TaxoDros database (Bächli, 2025); (iii) number of individuals and populations assessed, as well as their respective biogeographic regions; (iv) number, identity and inheritance patterns of the employed genetic markers; (v) whether the study included phenotype data; (vi) whether the study employed molecular clock to estimate divergence/diversification times; and (vii) whether the study employed Ecological Niche Modeling (ENM) as a complementary approach. After this, we performed a final filter step to exclude studies that either did not use molecular markers or included fewer than two populations. Biogeographical regions of the sampled species followed the regionalization proposed by Morrone (2015), and geological periods followed the scale proposed by Walker and Geissman (2022). Graphs were plotted in R 4.4.2 (R Core Team 2025) using the *ggplot2* package (Wickham, 2016).

## Results and Discussion

### General overview

In the initial Web of Science survey, we retrieved 4,169 articles, of which 3,602 were original papers (Table S1). When this dataset was submitted to ASReview screening, labeling of relevant papers clearly asymptoted after approximately 800 rounds (Figure S1a). However, we continued our manual inspection until round 2,050, in which the threshold of 300 consecutive non-relevant records was reached (Figure S1b). This process allowed us to reduce the number of papers to 312 (Table S2), of which 167 employed at least two populations and one molecular marker (Table S3). This dataset was then examined to evaluate temporal and journal-based patterns in the distribution of papers.

**Figure 1 -:**
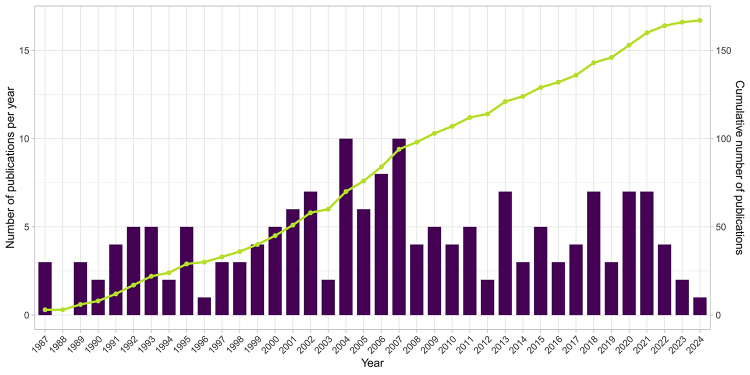
Number (left Y axis, purple bar plot) and cumulative number (right Y axis, green line plot) of phylogeographic and genetic structure articles employing Drosophilidae species per year, from 1987 to 2024.

The number of publications per year ranged from 0 (in 1988) to 10 (in 2004 and 2007) and showed a steady increase over the first 30 years (Figure 1), following the trend reported by Beheregaray (2008). After that, the number of studies per year stabilized at approximately five. The 167 papers evaluated were published across 48 journals, most of which have high impact factors, with a weighted Journal Citation Reports average score of 3.65 (Clarivate Analytics, 2024) (Table 1). The most frequent publication venues - “Genetics” and “Molecular Ecology” - accounted for more than one-quarter of all papers. Nevertheless, most journals have published only a single paper during the period analyzed, accounting for 13.77 % of the total retrieved publications. 

**Table 1 -:** List of journals that published the retrieved articles, with the corresponding number of recovered publications and Journal Citation Reports (Clarivate Analytics, 2024) scores.

Journal	No of articles	JCR (2024)
Genetics	25	5.1
Molecular Ecology	23	3.9
Molecular Biology and Evolution	13	5.3
Journal of Evolutionary Biology	8	2.3
Evolution	7	2.6
Genetica	7	1.3
Journal of Heredity	7	2.5
Biological Journal of the Linnean Society	6	1.5
Heredity	6	3.9
Molecular Phylogenetics and Evolution	5	3.6
Journal of Pest Science	4	4.1
PloS One	4	2.6
Biological Invasions	3	2.6
Ecology and Evolution	3	2.3
Mitochondrial DNA Part A	3	0.6
Biochemical Genetics	2	1.6
Genetics and Molecular Biology	2	1.3
Genome Biology and Evolution	2	2.8
Hereditas	2	2.5
Journal of Biogeography	2	3.6
Journal of Molecular Evolution	2	1.8
Journal of Zoological Systematics and Evolutionary Research	2	2.6
Proceedings of the National Academy of Sciences of the United States of America	2	9.1
Proceedings of the Royal Society B - Biological Sciences	2	3.5
Zoological Studies	2	1.4
Anais da Academia Brasileira de Ciências	1	1.1
Annales de la Societe Entomologique de France	1	0.7
Annals of the Entomological Society of America	1	1.8
Biotropica	1	1.7
BMC Ecology and Evolution	1	2.6
BMC Genetics	1	2.9
BMC Genomics	1	3.7
European Journal of Entomology	1	1.2
G3-Genes Genomes Genetics	1	2.2
Genetics Research	1	2.1
Genetics Selection Evolution	1	3.1
Genome	1	1.8
Genome Research	1	5.5
Genomics	1	3.0
Journal of Economic Entomology	1	2.4
Journal of Genetics	1	1.2
Journal of Insect Science	1	2.0
Nature	1	48.5
Nucleic Acids Research	1	13.1
PloS Genetics	1	3.7
Russian Journal of Genetics	1	0.5
Canadian Entomologist	1	1.1
Zoological Science	1	1.0
TOTAL	167	3.65*

*Weighted average of impact factor.

### Sampling bias at several taxonomic levels

**Figure 2 -:**
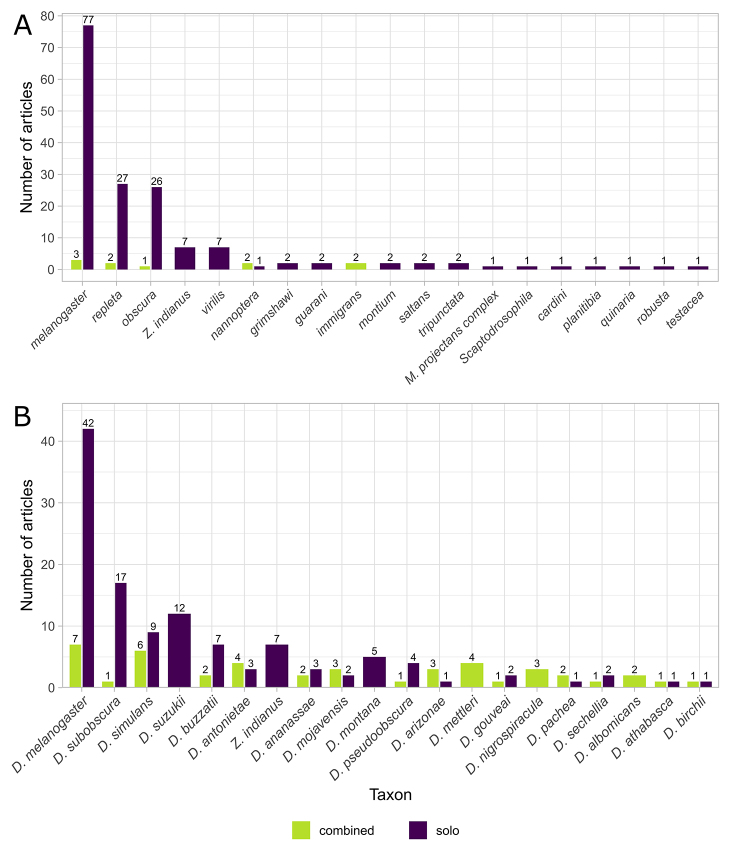
Total number of articles studying different Drosophilidae species groups (A) and the top 20 most studied species in the family (B). Green bars represent studies that focused on two or more taxa; purple bars represent studies that focused on a single species.

Of the 77 Drosophilidae genera (Bächli, 2025), only four have been studied in a phylogeographic or genetic structure context: *Drosophila* (158 studies), *Mycodrosophila* (1 study), *Scaptodrosophila* (1 study), and *Zaprionus* (7 studies) (Table S3). There is also a strong bias toward certain *Drosophila* species groups, with only 16 of the 59 recognized groups (Bächli, 2025) represented in these studies (Figure 2A; Table S4). The *melanogaster* group stands out as the most extensively studied (80 articles), followed by the *repleta* (29 articles) and *obscura* (27 articles) groups of *Drosophila* (Figure 2A). The three groups also include the highest numbers of evaluated species - 26, 10, and 8, respectively (Table S4). Therefore, at least 43 *Drosophila* species groups remain to be studied in a phylogeographical context, in addition to the thousands of species belonging to the different Drosophilidae genera. This pronounced bias is likely explained by a disproportionate research interest in *D. melanogaster* and related species, as well as by difficulties in sampling and identifying non-*Drosophila* species. Many of these species are, in fact, typically collected in small numbers or through occasional sampling and can be reliably identified only from male specimens by taxonomic specialists (Machado et al., 2017).

A marked bias was also observed in the identity of the investigated species, with only 28 being represented in more than one study. In this context, more than half (~57 %) of the recorded studies focused on just four species: 49 on *D. melanogaster*, 18 on *D. subobscura*, 15 on *D. simulans*, and 12 on *D. suzukii* (Figure 2B; Table S4). Among these, the strong interest in *D. melanogaster* clearly stems from its fundamental role in advancing our understanding of genetics and evolution (Markow and O’Grady, 2006). *Drosophila simulans*, in turn, is not only closely related to *D. melanogaster* (Finet et al., 2021) but has also achieved a worldwide distribution through a similar evolutionary history. Consequently, it is often used to compare genetic patterns with those observed for *D. melanogaster* (Singh et al., 1987; Begun and Aquadro, 1993; Verspoor and Haddrill; 2011; Machado et al., 2016). *Drosophila subobscura* is among the most extensively studied *Drosophila* species in Europe. Over the last 50 years, it has rapidly spread from the Palearctic into Nearctic and Neotropical regions, where it has developed clinal patterns for some chromosomal arrangements that parallel those observed in Old World populations (Prevosti et al., 1988). Finally, *D. suzukii* (also known as the spotted-wing *Drosophila*) is native to East and Southeast Asia but has become a major agricultural pest in the Americas and Europe since the late 2000s (Attallah *et al.*, 2014), causing significant economic losses to fruit crops.

### Molecular markers

**Figure 3 -:**
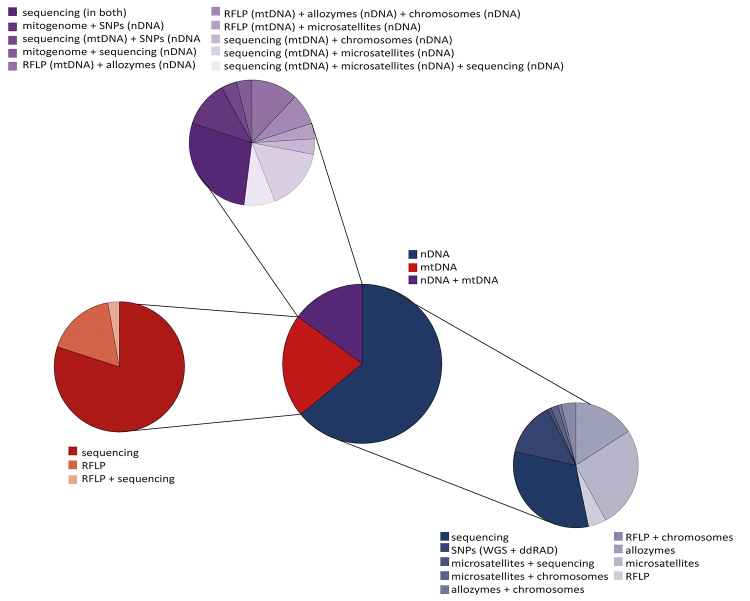
Proportion of articles employing different genetic markers, according to their corresponding genetic compartment. Blue, red, and purple pie charts represent studies using nuclear, mitochondrial, and combined genetic markers, respectively.

Considering inheritance patterns, most studies addressing phylogeography and genetic structure in Drosophilidae relied exclusively on nuclear markers (~64.1 %, 107 out of 167) (Figure 3; Table S5). Otherwise, only ~21 % (35 out of 167) of the studies focused solely on mitochondrial markers, and ~14.9 % (25 out of 167) combined both nuclear and mitochondrial markers. This pattern differs from that reported by Beheregaray (2008) and Turchetto-Zolet et al. (2013), whose literature surveys found that ~72 % and ~55 % of the studies, respectively, employed only mitochondrial markers. Although this incongruence may partially reflect the historical context of each survey, showing a gradual shift in marker preference, it probably also reflects taxon-specific patterns. Indeed, evolutionary research in Drosophilidae has a long tradition of using allozyme and RFLP markers, which together account for ~24.2 % of the studies using nuclear markers (32 out of 132). There is also a differential preference for microsatellite markers, employed in ~28.8 % of these studies (38 out of 132), likely reflecting the wide availability of microsatellite primers for some of the best-studied species in the *melanogaster*, *obscura*, and *repleta* groups, as well as their heterologous applications.

Concerning nuclear markers, phylogeography and genetic structure studies have, over the past decade, transitioned from using a few nuclear loci to exploring entire genomes - a shift that has given rise to the field of genomic phylogeography (reviewed in Brito and Edwards, 2009). This pattern can also be seen in Drosophilidae studies, where single-nucleotide polymorphisms (SNPs) characterized using Whole Genome Shotgun (WGS) strategies or reduced-representation approaches such as RAD-seq already represent the fourth most widely used class of nuclear markers. In fact, although Schlötterer and Harr (2002) were the first to employ SNPs to infer phylogeographic or population structure patterns in Drosophilidae, the first genome-wide SNP mapping conducted with this purpose was performed by Pool et al. (2012), followed by 17 additional studies over the last 12 years (Table S5). Only two of these cases used reduced-representation approaches based on RAD-seq (Koch et al., 2020; Nelson et al., 2023). 

As for nuclear markers, both historical traditions and recent advances likely contribute to explaining the observed patterns for mitochondrial markers. Among the 60 studies employing mitochondrial markers, 13 used RFLP to characterize restriction sites or fragment size from either the whole mitogenome or from specific mitochondrial genes (Table S5). Of the 43 articles analyzing mitochondrial gene sequences, cytochrome oxidase subunit I (COI) and cytochrome oxidase subunit II (COII) were the most commonly used genes, appearing in 26 and 13 studies, respectively. Only four studies employing mitochondrial markers used whole mitogenome sequences. 

Frequently, the patterns obtained for molecular markers were complemented or compared with those observed at the chromosomal and phenotypic levels. Specifically, 10 of the recovered articles examined chromosome rearrangements, whereas 16 analyzed phenotypic traits (Table S3). In the last case, evaluations involved external and internal morphology patterns, as well as behavioral and physiological data, ranging from geometric morphometry of wing landmarks (Moraes and Sene, 2007; Koch et al., 2020; Barrios-Leal et al., 2021; Machado et al., 2022) to mate choice tests (Schug et al., 2008; Yukilevich et al., 2018). Nevertheless, although strategies associated with Environmental Niche Modelling (ENM) have become common as a complementary approach to addressing biogeographic patterns and demographic history across time in phylogeographic studies using information independent of genetic data (Knowles, 2009), they were employed in only six of the evaluated studies.

### Biogeographic regions

**Figure 4 -:**
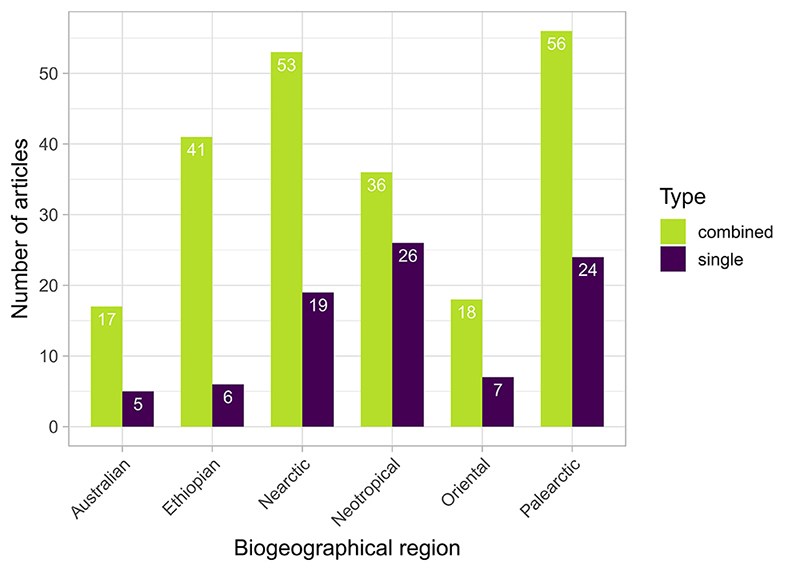
Distribution of retrieved articles according to the biogeographical region of sampled populations, following Morrone (2015). Green bars represent studies including specimens sampled in two or more regions; purple bars represent studies that sampled specimens in a single region.

The Palearctic region was represented in 80 of the 167 studies, making it the most frequently investigated biogeographical region in the dataset (Figure 4). The Nearctic and Neotropics follow as the second and third-most-studied regions, with 72 and 62 papers, respectively. In contrast, the Ethiopian, Oriental, and Australasian regions appear in 47, 25, and 22 studies, respectively. Interestingly, in most studies (87 out of 167), species were sampled across more than one biogeographic region, reflecting a prevalence of studies involving broadly distributed species rather than endemic ones.

### Palearctic region

According to Morrone (2015), the Palearctic region comprises Europe, North Africa, and most of temperate Asia, extending from the Atlantic coasts to eastern Siberia and the Japanese archipelago. Of the 80 studies involving the Palearctic region, 38 were dedicated to solving different aspects of the evolutionary history of *D. melanogaster*, especially those related to its origin and demographic history (see, for example, Baudry et al., 2004; Arguello et al., 2019; Sprengelmeyer et al., 2020), or population structure and admixture levels (see, for example, Pool et al., 2012; Kao et al., 2015). Demographic analysis performed by Sprenhelmeyer *et al.* (2020) suggests that populations of this species expanded from south-central Africa ~13 kya, but reached Europe much later, at 1.8 kya. 

In addition, 14 papers have focused on Palearctic populations of *D. subobscura*, most of which investigated the species’ introduction history into other biogeographic regions (Rozas et al., 1995; Pinto et al., 1997; Pascual et al., 2007; Araúz et al., 2011). According to Araúz *et al.* (2011), the colonization of the American continent by *D. subobscura* was most probably a single event involving colonizers from the western Mediterranean region. Otherwise, Brehm et al. (2004) evaluated population structure across Moroccan and Iberian populations of the species, rejecting the hypothesis that the Strait of Gibraltar has acted as a barrier to gene flow, and dating radiation to the Canary Islands to approximately 1.1 Mya.

*Drosophila suzukii* is another well-studied species in the Palearctic, where considerable effort has been devoted to investigating its colonization history (Lavrinienko et al., 2017). In this context, Adrion et al. (2014) supported that invasions of Europe and the continental United States are independent demographic events associated with strong bottleneck signatures. Otherwise, Lewald et al. (2021) showed that European and Nearctic populations of *D. suzukii* encompass two distinct clades, suggesting independent migrations from Asia. They also detected signals of population admixture from Western United States populations back to Asia.

In addition to the aforementioned *Drosophila* species, considerable attention (seven recorded studies) has also been given to Palearctic populations of *D. montana* and *D. virilis*, both belonging to the *virilis* group. According to Mirol et al. (2007), North American and European populations are clearly differentiated, with divergence dating back to the Pleistocene. Otherwise, Mirol *et al.* (2008) suggest a recent worldwide exponential expansion of the species associated either with post-glacial colonization or with domestication. Another interesting study involved a comparative phylogeographical study between three species of *Drosophila* [*D. albomicans* (*immigrans* group), *D. bipectinata* and *D. takahashi* (*melanogaster* group)], and one species of a *Drosophila*-parasitoid wasp (*Leptopilina ryukyuensis*). Specific phylogeographical patterns were found for each species, thus suggesting no correlation between host and parasite evolutionary histories. In this case, highly differentiated lineages were detected for *D. formosana* and *D. immigrans*, whose divergence was associated with Pliocene and Pleistocene interglacial events.

### Nearctic region

The Nearctic region comprises most of North America, including Canada, the United States, and northern Mexico (Morrone, 2015). In total, phylogeographical studies in this region included 36 species belonging to the *melanogaster*, *nannoptera*, *obscura*, *quinaria*, *repleta*, *testacea*, *tripunctata*, and *virilis* groups of *Drosophila*, as well as *Zaprionus indianus* (Table S3). Nevertheless, once again, cosmopolitan, exotic, and invasive species such as *D. melanogaster*, *D. simulans*, *D. subobscura*, *D. suzukii*, and *Z. indianus* accounted for the majority of studies (45 out of 72).

Regarding *Z. indianus*, Comeault et al. (2021) suggested a single expansion from Africa to the western hemisphere, since Nearctic and Neotropical populations are genetically more similar to each other than to African lineages. Nonetheless, they also showed that populations from ancestral and introduced ranges exhibit shared patterns of genetic diversity that are consistent with parallel selection. Interestingly, signals of parallel differentiation have also been reported between northern and southern populations of Australian and North American populations of *Drosophila simulans* (Sedghifar et al., 2016), underscoring the significant influence of regional environmental heterogeneity on the evolutionary dynamics of Drosophilidae species. In this sense, clines of latitudinal variation have already been reported for this region in different species, such as *D. melanogaster* and *D. simulans* (Costa et al., 1992; Machado et al., 2016) and *D. montana* (Wiberg et al., 2021).

Studies on native cactophilic species of the *repleta* group - including *D. mojavensis*, *D. arizonae*, *D. mettleri*, *D. nigrospiracula*, *D. navojoa*, *D. mainlandi*, *D. hamatofila*, *D. aldrichi*, and the *D. anceps* species complex - have also revealed shared patterns of population differentiation and demographic history (Markow et al., 2002; Hurtado et al., 2004; Machado et al., 2007). These studies consistently identified common phylogeographical patterns across the Sonoran Desert (southwestern USA and northwestern Mexico), shaped by host specialization, recent population expansions, and geographic differentiation. Geographic barriers across the Nearctic appear to play a key role in structuring genetic diversity in different species. Notable examples include the separation of Baja California from the mainland, which occurred 3-6 Mya (Lonsdale, 1989) and constrained the dispersal of *D. pachea* (Markow *et al.*, 2002; Hurtado *et al.*, 2004); and the Sierra Madre Occidental mountain range and the Trans-Mexican volcanic belt, which divide western and eastern, as well as northern and southern populations of *D. arizonae* (Machado *et al.*, 2007; Rampasso et al., 2017). In some species, high levels of population structure have suggested the presence of geographic host races and incipient speciation (Rampasso *et al.*, 2017). Evidence for recent population expansions (Hurtado *et al.*, 2004; Pfeiler et al., 2007; Pfeiler, 2019) has been linked to climatic oscillations that affected the Sonoran Desert during the Pleistocene (Van Devender, 2002).

### Neotropical region

The Neotropics - encompassing southern Mexico, Central America (with the Caribbean islands), and South America (Morrone, 2015) - are widely recognized as the most biodiverse region on the planet (Raven et al., 2020). This diversity is reflected in the distribution of phylogeographic studies across various *Drosophila* groups, with representatives of the *cardini*, *guarani*, *melanogaster*, *obscura*, *repleta*, *saltans*, *tripunctata*, and *virilis* groups, as well as the genera *Mycodrosophila* and *Zaprionus*, being the focus of at least one recorded study. Notably, native species of the cactophilic *repleta* group account for ~37 % of the studies (23 out of 62), whereas those from the *melanogaster* group account for ~30 % (19 out of 62). Consequently, only 20 studies are distributed among the other lineages.

Several studies have shown that the current distribution of Drosophilidae populations in this region has been severely influenced by the climatic oscillations of the Pleistocene and Holocene, as well as by complex geographical and ecological barriers that fostered isolation, diversification, and the formation of regional refugia. Within this historical and environmental context, several interesting outcomes have emerged from studies on cactophilic species, such as the *D. buzzatii* cluster (Barrios-Leal et al., 2019; Santos et al., 2023) and the *D. serido* and *D. antonietae* haplogroups (Franco and Manfrin, 2013). In both cases, vicariant events, demographic fluctuations, and migratory routes were widely affected by Quaternary paleoclimatic changes on the spatial dynamics of the Seasonally Dry Tropical Forests (SDTFs) (Santos *et al.*, 2023). This has led to shared patterns of distribution and divergence, as detected, for example, in the *D. antonietae* and *D. serido* clades, whose diversification was associated with the fragmentation of the SDTF during interglacial periods (Franco and Manfrin, 2013). Otherwise, whereas Pleistocene paleoclimatic oscillations appear to have led to a range expansion from Chaco to Cerrado and Catinga in *D. buzzatii* (Barrios-Leal *et al.*, 2019), they were associated with a deep population structure among mainland and coastal populations of *D. meridionalis* (Barrios-Leal *et al.*, 2018). In fact, responses of the *repleta* group species to climatic oscillations varied among species, possibly due to differences in ecology, distribution, and evolutionary potential imposed by genetic constraints. 

Variations in ecological requirements also shaped distinct responses to Pleistocene climatic oscillations: whereas dry environment species expanded their populations during glacial periods, those of humid habitats exhibited the opposite pattern. A complementary perspective on this pattern comes from studies involving *D. maculifrons* and *D. ornatifrons*, two humid forest species that exhibit signs of population expansion during Pleistocene interglacial periods (De Ré et al., 2014; Gustani et al., 2015). These expansions coincide with increases in temperature and humidity in southern and southeastern Brazil, which promoted the expansion of forested areas (Carnaval et al., 2009). Interestingly, several of these conclusions were reached through the combined use of molecular markers and Environmental Niche Models (ENM), whose concordant results certainly provided greater confidence to the inferred patterns (De Ré *et al.*, 2014; Barrios-Leal et al., 2019).

### Ethiopian region

The Ethiopian region comprises sub-Saharan Africa, the island of Madagascar, the northern Arabian Peninsula, and the West Indian Ocean islands (Morrone, 2015). Studies in this region included species belonging to the *melanogaster* and *obscura* groups of *Drosophila*, and *Zaprionus indianus* (Table S4). Nevertheless, the bias toward studies involving the *melanogaster* group is even more pronounced in this region, where these species were the focus of approximately 94 % of the recorded papers (44 out of 47 studies). In fact, the African continent has been consistently identified as the ancestral region of the cosmopolitan *D. melanogaster* (Baudry et al., 2004; Arguello et al., 2019) and the invasive *Z. indianus* (Markow et al., 2014; Comeault et al., 2021), which has motivated most phylogeographic and genetic structure studies in this region.

Despite the agreement involving the origin of *D. melanogaster* in the Ethiopian region, the precise location on the continent remains a matter of debate. Some authors have suggested that this occurred either in the Eastern (Benassi and Veuille, 1995; Baudry et al., 2004) or the Western Africa (Dieringer et al., 2005). Nevertheless, recent studies using genomic data have provided strong support for the hypothesis that *D. melanogaster* originated in the region of Austral Africa, in the territories currently comprising Zambia and Zimbabwe (Verspoor and Haddrill, 2011; Pool et al., 2012; Sprengelmeyer et al., 2020). The range expansion within Africa is estimated to have begun at least 72 kya, as dated for the divergence between Austral and Western populations (Kapopoulou et al., 2018). In contrast, the spread of *D. melanogaster* beyond the Ethiopian region - often referred to as the single “out of Africa” event (Baudry *et al.*, 2004) - appears to have occurred between 12 and 19 kya (Li and Stephan, 2006; Arguello et al., 2019), and was followed by severe bottlenecks that substantially reduced the genetic diversity of non-African populations (Begun and Aquadro, 1993; Haddrill *et al.*, 2005). Finally, there is also compelling evidence suggesting that some populations subsequently migrated back to Africa (Benassi and Veuille, 1995; Pool *et al.*, 2012; Arguello *et al.*, 2019; Sprengelmeyer *et al.*, 2020; Coughlan et al., 2022).

Concerning *Z. indianus*, Comeault et al. (2021) showed that introduced populations in South America and North America carry a genomic signature of a common range expansion, and were colonized by the western African lineage following a major admixture event between western and eastern African lineages. According to the best-fit demographic scenario, this split occurred ~600 generations (~35-60 years ago), which agrees with the first records of the species in the American continent around 1990 (van der Linde et al., 2006). Accordingly, Mattos Machado et al. (2005) have evidenced the absence of genetic structure among Brazilian populations, supporting the hypothesis of a single colonization event. Otherwise, these authors also suggested a moderately sized propagule was introduced to Brazil, since invading populations were only slightly less diverse than ancestral ones. In America, northward migration of individuals from Brazil would have led to the spread of the species into Central America and Mexico (Markow et al., 2014) or even into Argentina (Fernandez Goya et al., 2020). 

### Oriental region

The Oriental region encompasses the tropical areas of Eurasia and Southeast Asia, including India, the Himalayas, Myanmar, Malaysia, Indonesia, the Philippines, and the islands of Micronesia, Polynesia, and Hawaii (Morrone, 2015). Phylogeographical studies of Drosophilidae in this region have also been strongly focused on the *melanogaster* group (18 of 25 studies), although some studies also evaluated endemic species (e.g., the *grimshawi* and *planitibia* groups). 

Several studies have revealed patterns of population expansion and genetic structure in this biogeographic region (Das et al. 2004; Schug et al. 2007, 2008). Das *et al.* (2004), for example, traced the migration route of *D. ananassae* to a landmass called “Sundaland” in Southeast Asia during the Pleistocene (about 18 kya), when sea levels were lower. This region harbors five central populations of the species that exhibit high levels of nucleotide diversity and low linkage disequilibrium (Das *et al.* 2004), which are typical of ancestral populations. Migration from Sundaland appears to have occurred in a radial pattern, while subsequent patterns of dispersion in peripheral populations (in Asia and the South Pacific) possibly reflect historical human migratory routes (Das *et al.* 2004; Schug *et al.* 2007). In some cases, this history may have led to the evolution of distinct lineages or (incipient) cryptic species (Schug *et al.* 2007, 2008), some of which even exhibit some level of mating discrimination (Schug *et al.* 2008).

Interesting studies have also been conducted with Hawaiian species of the *grimshawi* group. For instance, Muir and Price (2008) compared the patterns of genetic diversity between populations of *D. engyochracea*, known from only two locations, and *D. hawaiiensis*, which is widespread across the archipelago. Both species were sampled from two isolated forest patches (*kipuka*). As expected, the more widely distributed *D. hawaiiensis* exhibited higher genetic diversity than the narrowly distributed *D. engyochracea*, although higher levels of gene flow were suggested for the latter. Moreover, there were some signals of mitochondrial introgression, evidenced by individuals morphologically identified as one species that carried haplotypes diagnostic of the other.

### Australian region

According to Morrone (2015), the Australian region comprises Australia, New Caledonia, New Guinea, New Zealand, and other Oceanian islands. Studies in this region included species belonging to the *immigrans*, *melanogaster*, *montium*, *obscura*, and *repleta* groups of *Drosophila*, as well as the *Scaptodrosophila* genus (Table S3). Nevertheless, ~64 % of these studies focused on species of the *melanogaster* group.

Among cosmopolitan species, the peripheral populations of *D. ananassae* present in Australia and other South Pacific locations exhibit evidence of migration from Southeast Asia during the last glacial maximum (Das et al., 2004; Schug et al., 2008). Strong support comes from microsatellite data, which revealed a complex pattern involving isolation-by-distance, pronounced population structure, range expansion, and multiple colonization events in some receptor populations (Schug *et al.*, 2007). On the other hand, neither population structure nor isolation-by-distance was observed in Australian populations of *D. melanogaster*, likely reflecting their shared history of recent colonization (Agis and Schlötterer, 2001).

Some interesting comparative phylogeography studies were also performed for species occurring in this biogeographic region. For example, in the tropical forests of eastern Australia, contrasting phylogeographic patterns were evidenced for the pair of sister species *D. serrata* and *D. birchii* (*melanogaster* group). The first - a generalist, widely distributed species - harbors two highly divergent and geographically structured mtDNA lineages, whereas the second - a specialist species, restricted to rainforests - shows no significant population structure (Kelemen and Moritz, 1999). Two main hypotheses, not mutually exclusive, were proposed to explain the phylogeographical break in *D. serrata*: the splitting of populations into two distinct refugia during glacial periods, followed by secondary contact; or the formation of a pair of cryptic species. As for *D. birchii*, the low diversity and the lack of a geographical structure allowed hypothesizing a population expansion, which was later confirmed by Schiffer et al. (2007). Another interesting comparative study examined the endemics *Scaptodrosophila hibisci* and *S. aclinata*, which reproduce in flowers of *Hibiscus* (McEvey and Barker, 2001) and appear to have diverged following a strong bottleneck in eastern Australia about 40 kya (Barker, 2005). In this case, *S. aclinata* exhibited lower genetic variability, whereas *S. hibisci* exhibited signals of population expansion (Kelemen and Moritz, 1999; Barker, 2005).

Another interesting set of studies involved the neotropical cactophilic *Drosophila buzzatii*, a notorious invasive Drosophilidae species in the Australian region. This invasion occurred in the decade of 1930, when the host plant *Opuntia* was introduced in eastern Australia, leading *D. buzzatii* populations into a strong bottleneck event, with an estimation of 30-40 founders (Piccinali et al., 2007; Barker et al., 2009; Barker, 2013). Subsequent reductions in the distribution of the invasive plant resulted in new bottlenecks and population differentiation (Barker *et al.*, 2009; Barker, 2013). Microsatellites and the α-esterase5 gene, in particular, revealed notable changes in haplotype frequencies between colonizing and Argentine populations, confirming the strong founder effect (Frydenberg et al., 2002; Piccinali *et al.*, 2007).

## Conclusions and perspectives

Our comprehensive review of phylogeographic and genetic structure studies in Drosophilidae identified substantial research gaps, particularly regarding understudied taxa and biogeographical regions. The straightforward focus on certain species or species groups underscores the potential of phylogeographic approaches to shed light on overlooked dimensions of biodiversity. Moreover, the uneven distribution of research efforts across biogeographic regions highlights the opportunity to uncover novel phylogeographic patterns and evolutionary processes. After all, these regions may hold critical insights not only into the evolutionary histories of focal species but also into broader patterns shaping regional biodiversity. Finally, our findings reinforce the importance of Drosophilidae as model organisms in evolutionary and biogeographic research. Continued use of drosophilids in such efforts offers a powerful framework for advancing our understanding of biodiversity dynamics at both local and global scales.

## Supplementary Material

The following online material is available for this article:

Figure S1 – Performance of model training and automatic screenings on ASReview.

Table S1 – Complete set of original articles retrieved by the search string in Web of Science.

Table S2 – Set of articles that passed the automatic screening of ASReview under a machine learning framework.

Table S3 – Final set of articles that passed the last manual screening step, along with their corresponding attributes regarding the studied species and species groups, sampling design (number of populations and evaluated specimens), biogeographical regions, and genetic markers

Table S4 – Summary of the number of articles retrieved for each species, along with their corresponding taxonomic positioning.

Table S5 – Summary of the number of articles employing different genetic markers, according to their corresponding genetic compartment

## Data Availability

The datasets analyzed and/or generated during the current study are published in the article and available in the Supplementary Material section
