# Supplementary material for: Global patterns, biases, and advances in phylogeographic and genetic structure studies in Drosophilidae (Insecta: Diptera)
Source: Genet Mol Biol. 2026 Feb 9;49(Suppl 1):e20250246. doi: 10.1590/1678-4685-GMB-2025-0246 (PMC12895237; doi:10.1590/1678-4685-GMB-2025-0246)
Supplement: Figure S1 – [file 1415-4757-GMB-49-s1-e20250246-s1.pdf]

Genetics and Molecular Biology
https://doi.org/10.1590/1678-4685-GMB-2025-0246

Supplementary Material to "Global patterns, biases, and advances in phylogeographic and genetic structure studies in Drosophilidae (Insecta: Diptera)"

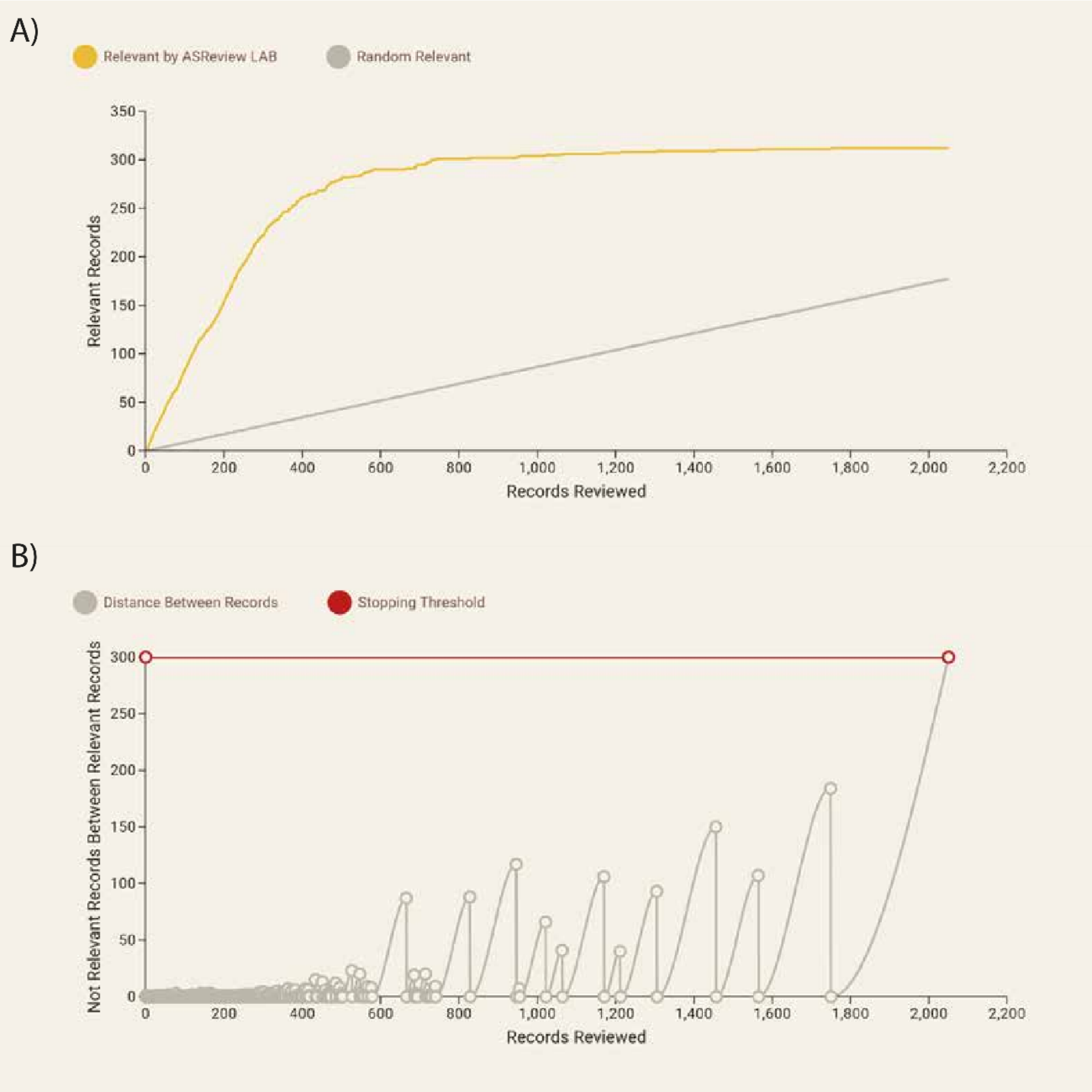

Figure S1 – Performance of model training and automatic screenings on ASReview. (A) Cumulative number of relevant records identified by the model (yellow) in comparison with random selection (gray). (B) Distance between consecutive relevant records across iterations (gray) and the stopping threshold (red).
